# Supplementary material for: Two-dimensional amine and hydroxy functionalized fused aromatic covalent organic framework
Source: Commun Chem. 2020 Mar 6;3:31. doi: 10.1038/s42004-020-0278-1 (PMC9814683; doi:10.1038/s42004-020-0278-1)
Supplement: Supplementary file 1 — Supplementary Information [file 42004_2020_278_MOESM1_ESM.pdf]

Supplementary Information

Two-dimensional amine and hydroxy functionalized fused aromatic covalent organic framework

By Mahmood et al.

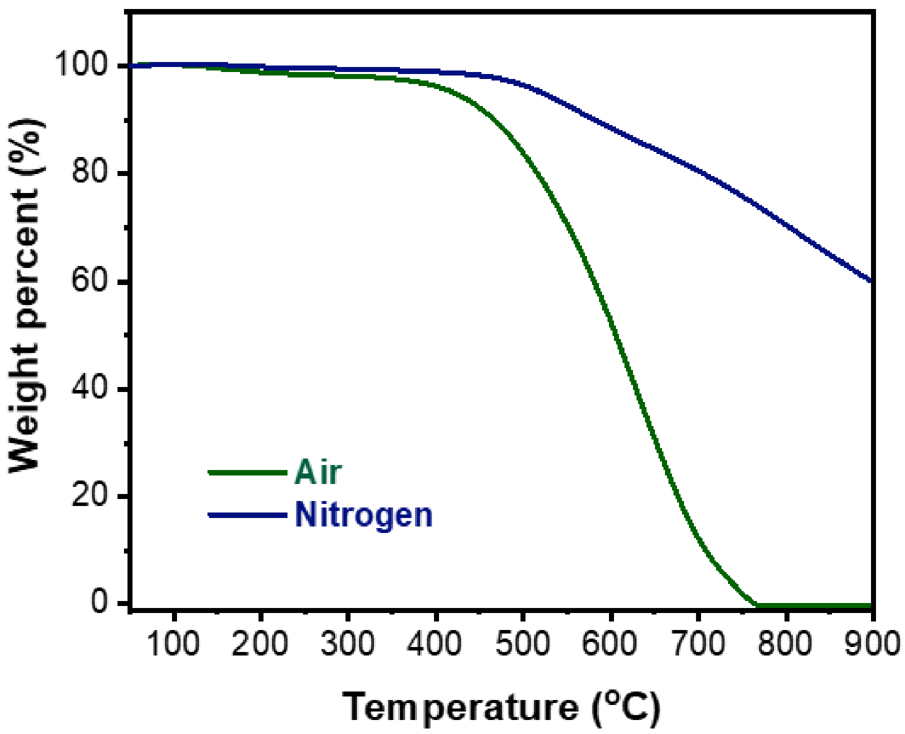

Supplementary Fig. 1. TGA thermograms of F-COF obtained with a ramping rate of 10 °C/min in air and nitrogen.

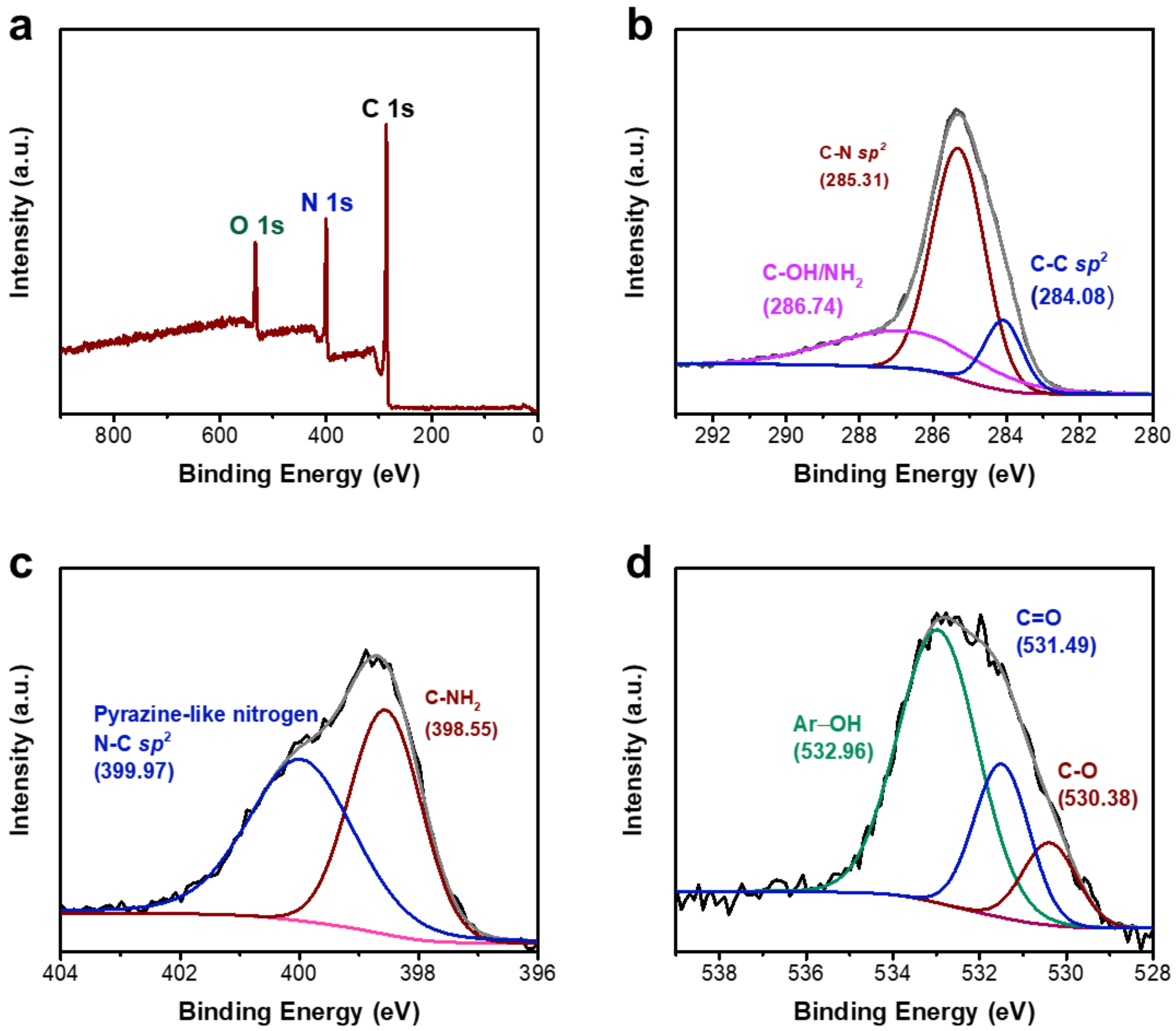

Supplementary Fig. 2 **a** XPS survey spectrum of F-COF. Deconvoluted XPS spectra: **b** C 1s; **c** N 1s; **d** O 1s.

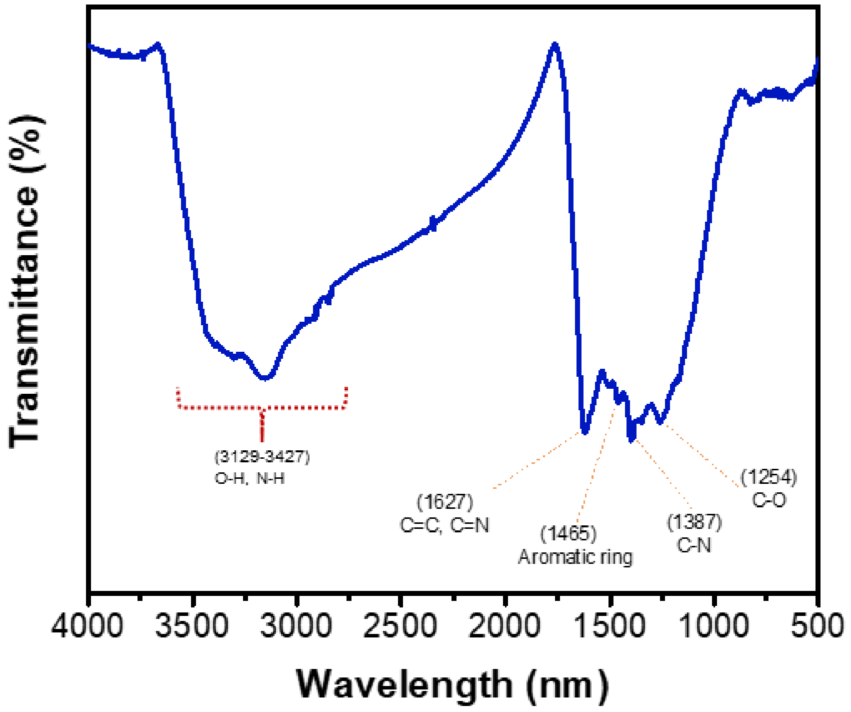

Supplementary Fig. 3. Fourier-transform infrared spectroscopy (FT-IR) spectrum of F-COF.

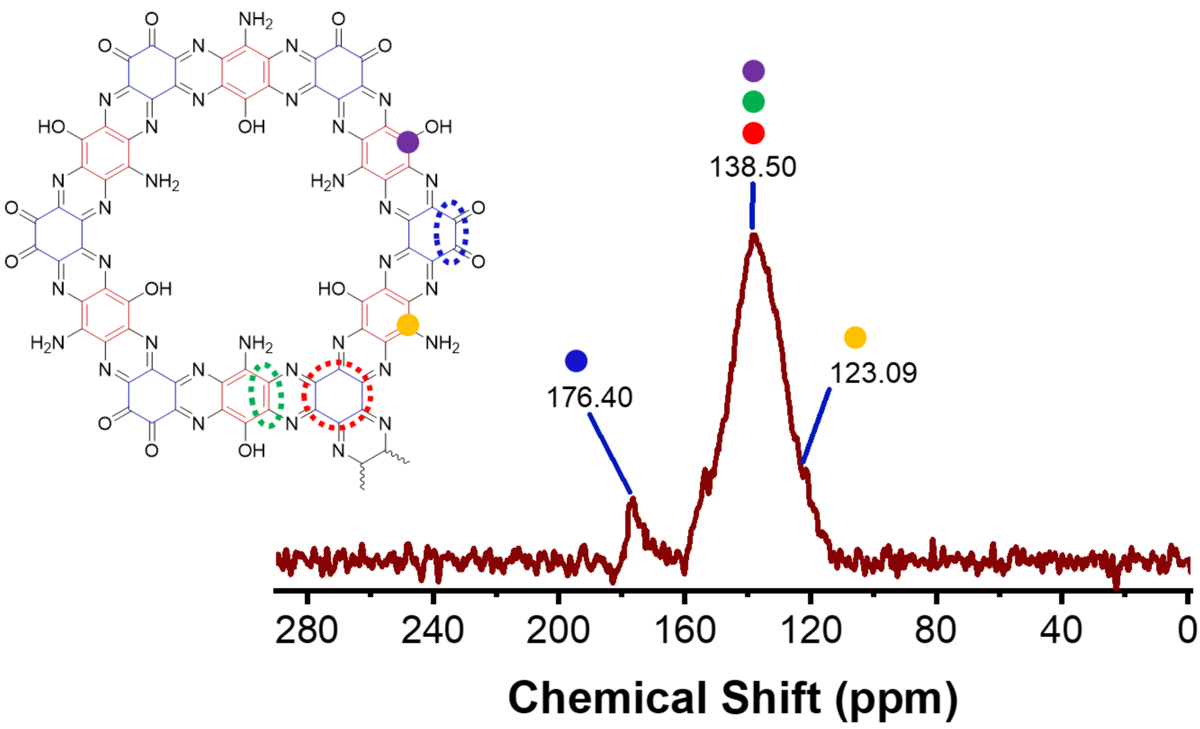

Supplementary Fig. 4. Solid-state carbon thirteen cross polarization-magic angle spinning nuclear magnetic resonance (^{13}C CP-MAS NMR) spectrum of F-COF.

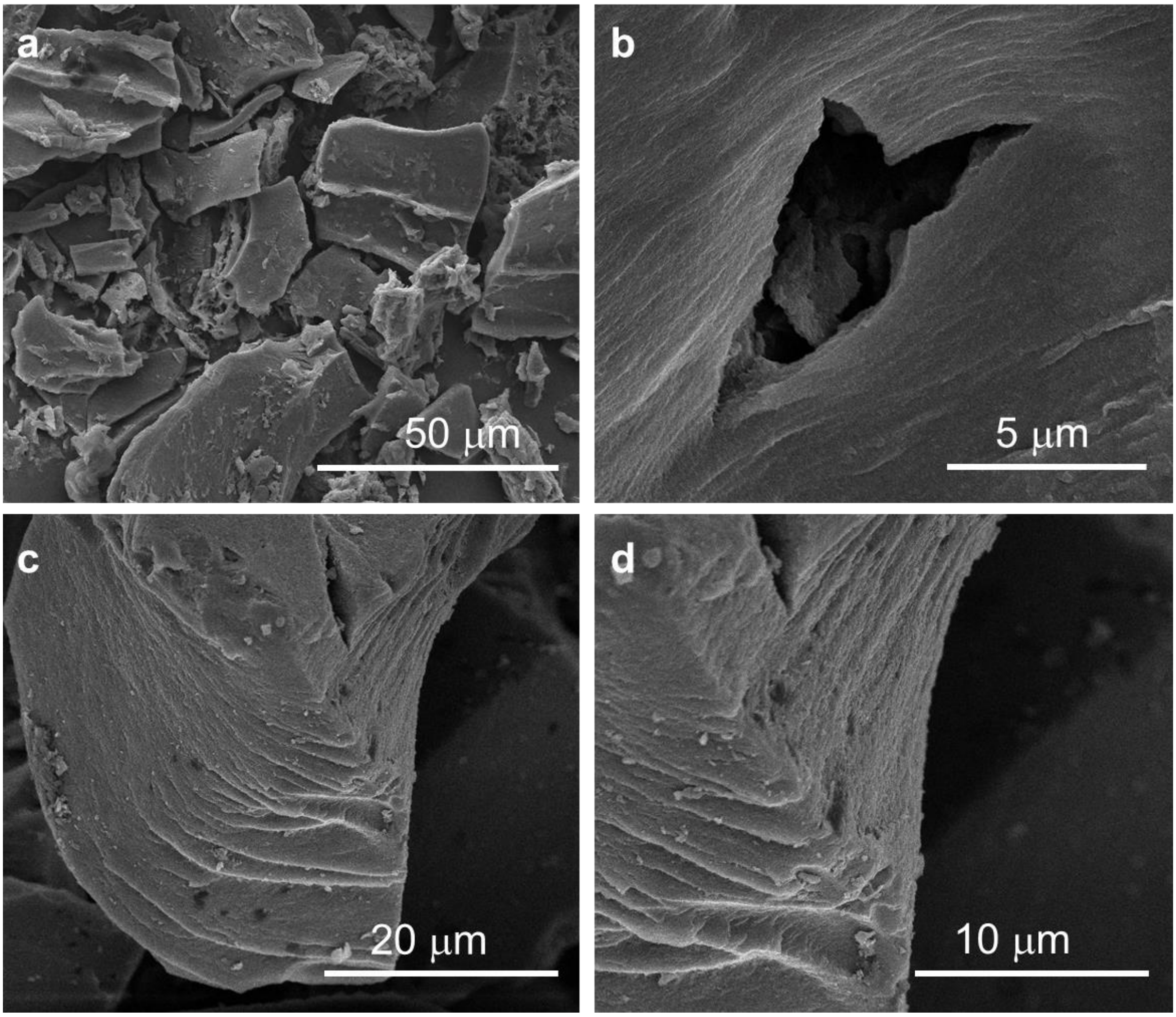

Supplementary Fig. 5. Scanning electron microscopy (SEM) images of F-COF at different magnifications: **a** ×1300; **b** ×10000; c ×3000; d ×6000

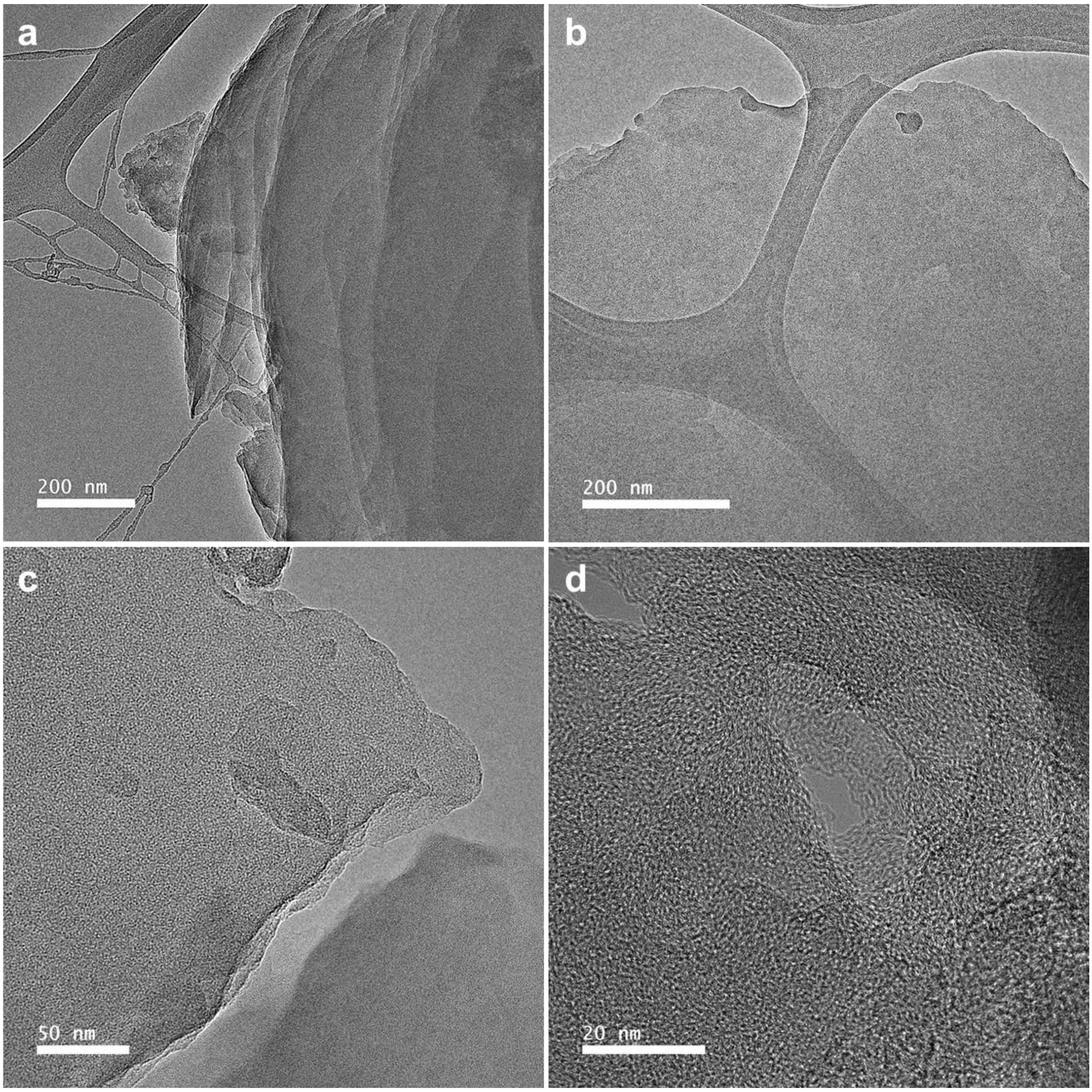

Supplementary Fig. 6. **High resolution transmission electron microscopy images of F-COF: a-b** Low magnification; **c-d** High magnification**.**

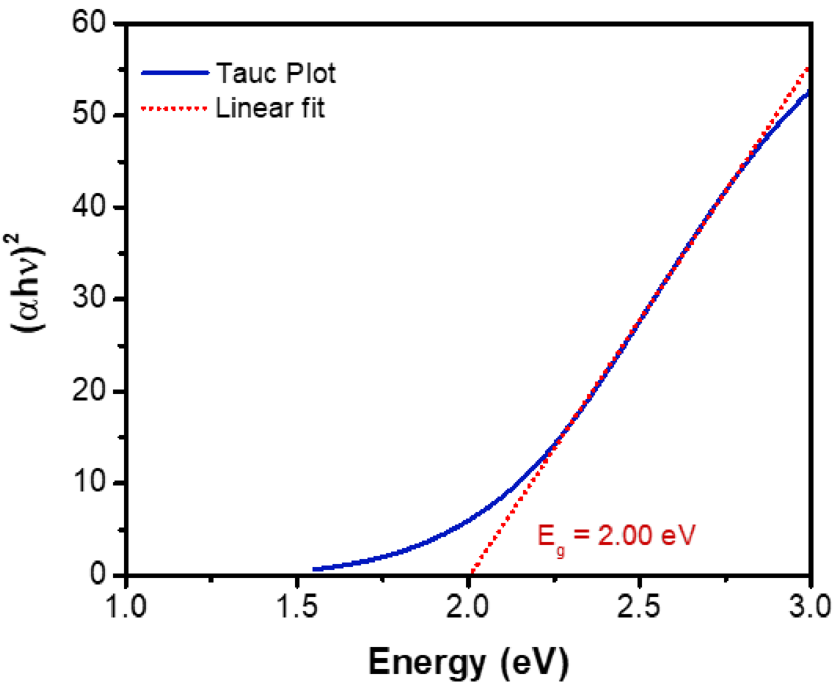

Supplementary Fig. 7. Tauc plots of F-COF obtained from absorbance data.

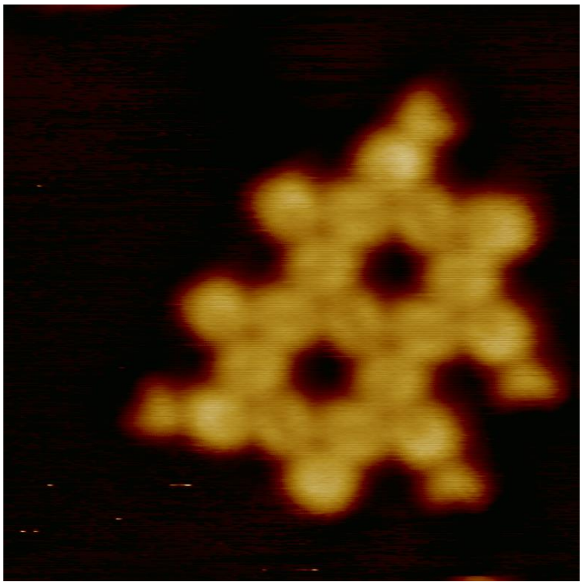

Supplementary Fig. 8. STM image of F-COF structure containing two holes (6.3 × 6.3 nm^2) obtained at a sample bias of –0.2 V and a tunneling current of 20 pA.

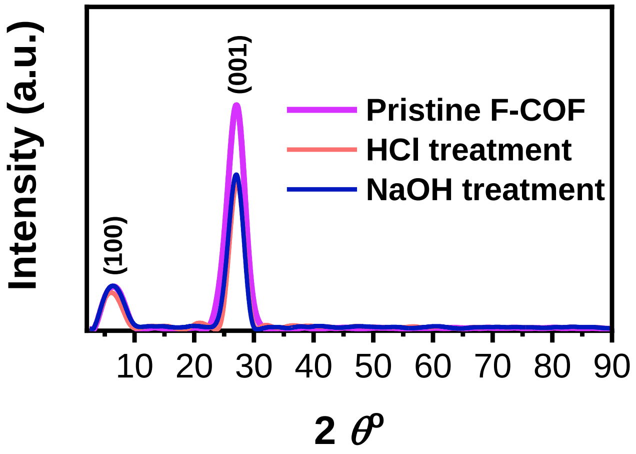

Supplementary Fig. 9. PXRD patterns of the F-COF before and after treatments with 12 M aq. HCl and 7 M aq. NaOH solutions for 24 hours.

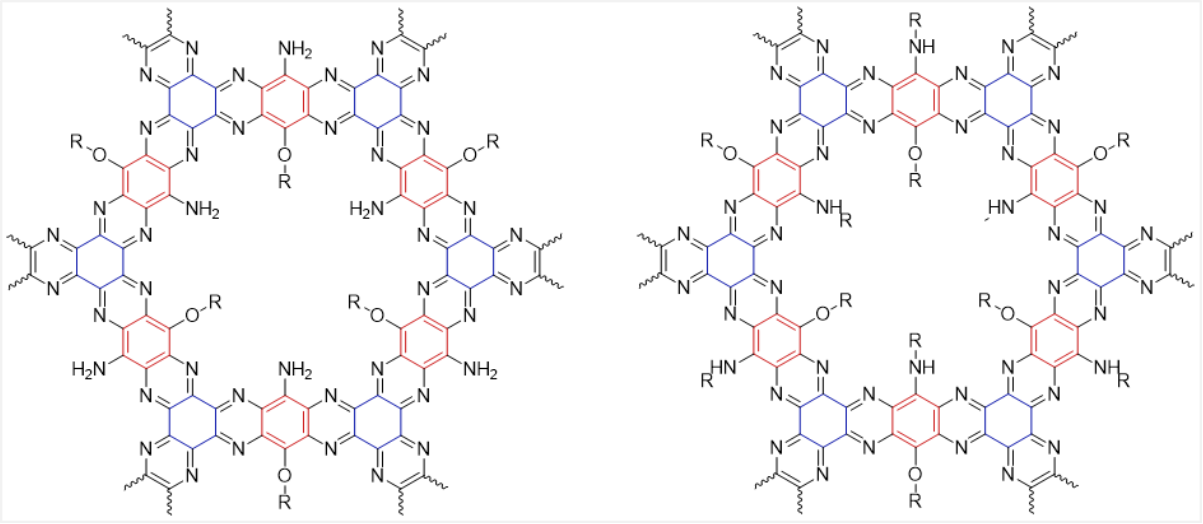

Supplementary Fig. 10. Possibility of the functionalization of (–NH_2 and –OH) functional groups.

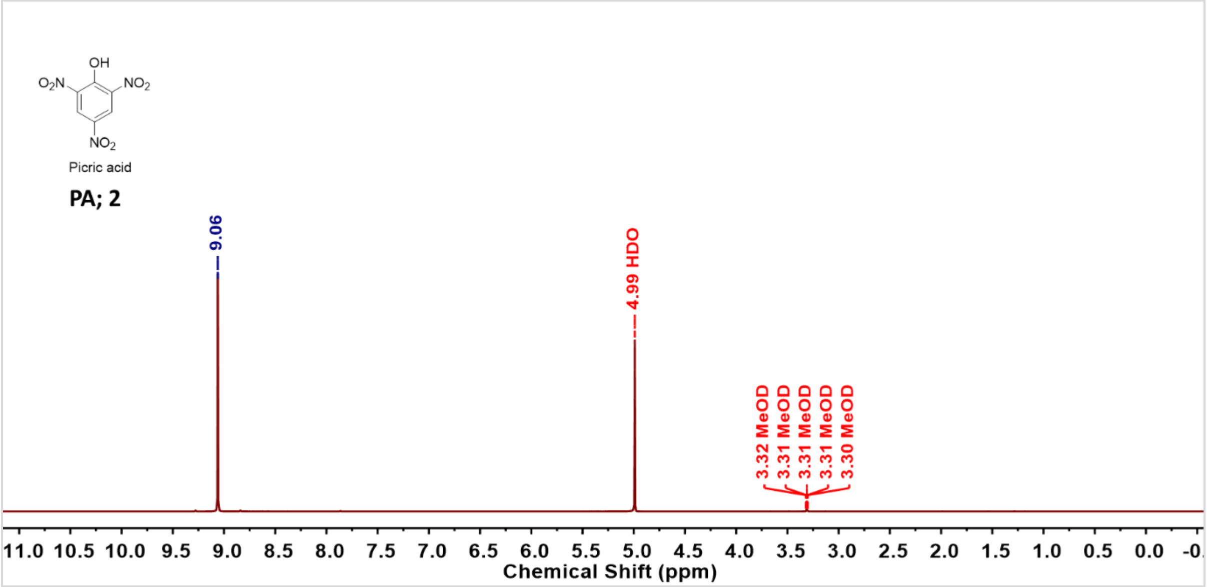

Supplementary Fig. 11. ^{1}H NMR spectrum of picric acid (PA; **2**) (MeOD, 400 MHz) after recrystallization from benzene.

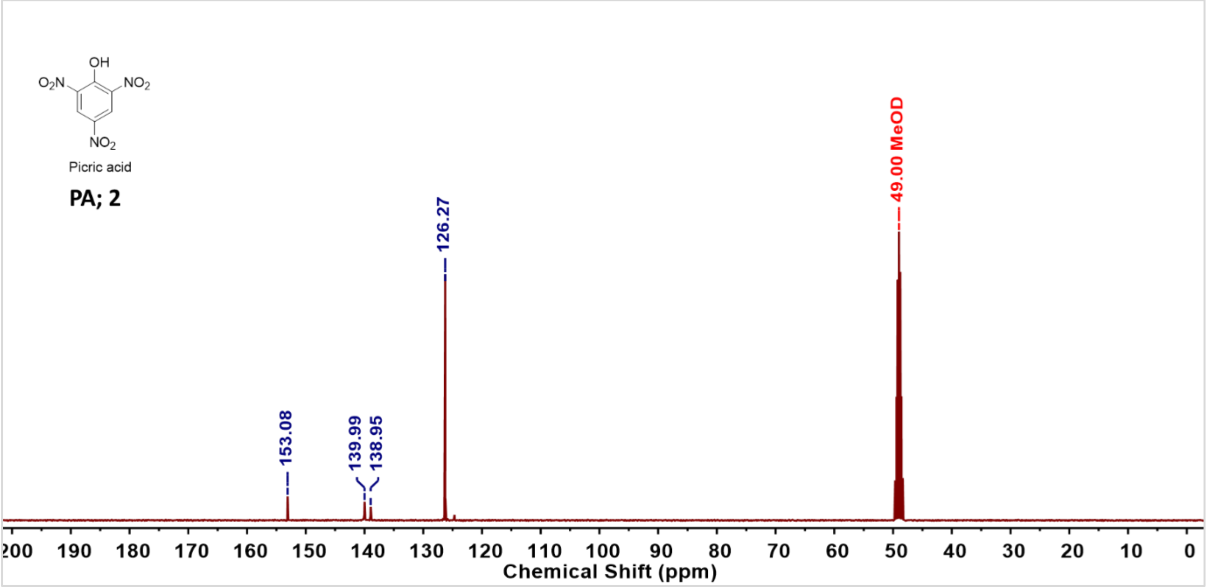

Supplementary Fig. 12. ^{13}C NMR spectrum of picric acid (PA; **2**) (MeOD, 400 MHz) after recrystallization from benzene.

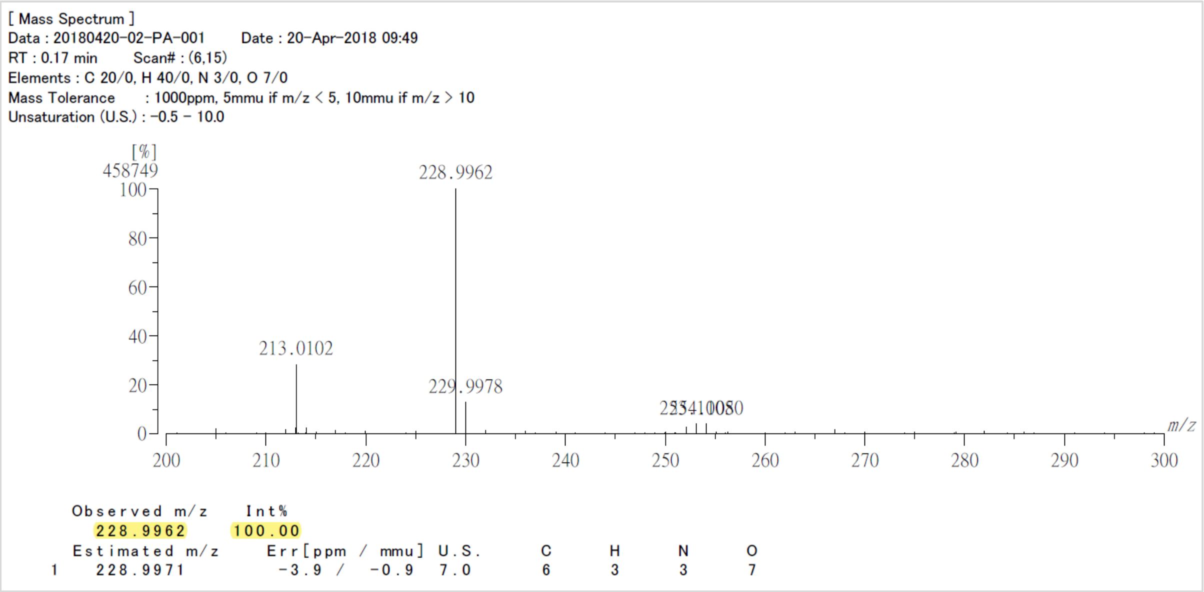

Supplementary Fig. 13. High-resolution mass spectrum (HR-MS) of picric acid (**PA; 2**) after recrystallization from benzene.

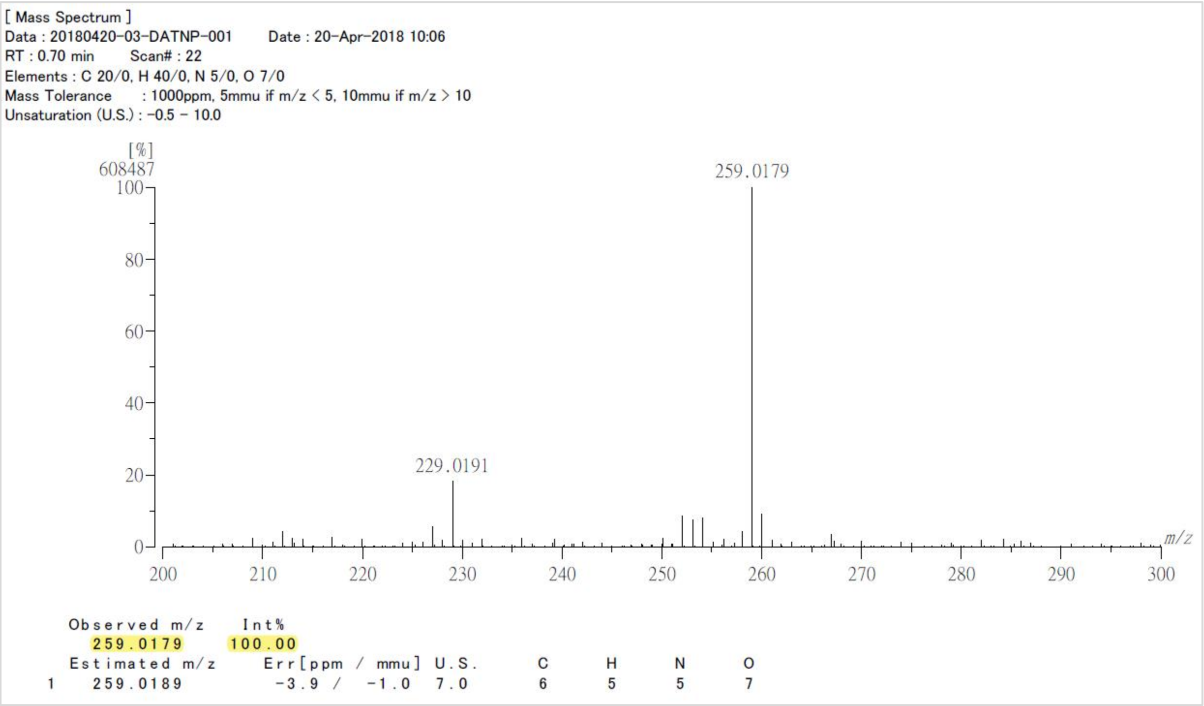

Supplementary Fig. 14. High-resolution mass spectrum (HR-MS) of 3,5-diamino-2,4,6-trinitrophenol (**DATNP; 3**).

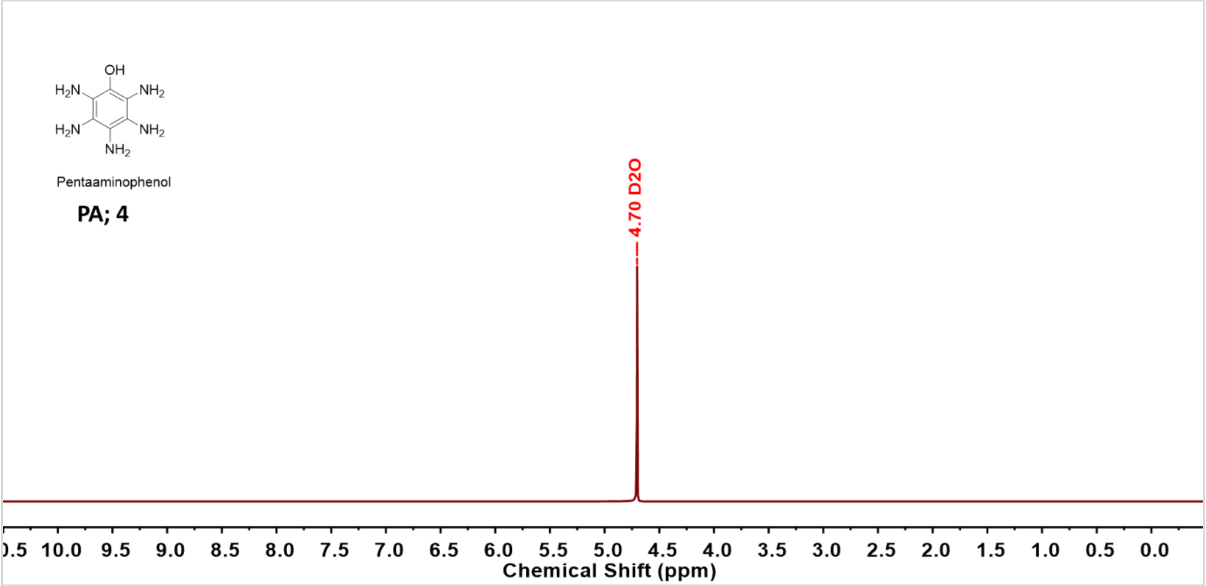

Supplementary Fig. 15. ^{1}H NMR spectrum of pentaaminophenol (PAP; **4**) (D_2O, 400 MHz) after recrystallization from benzene.

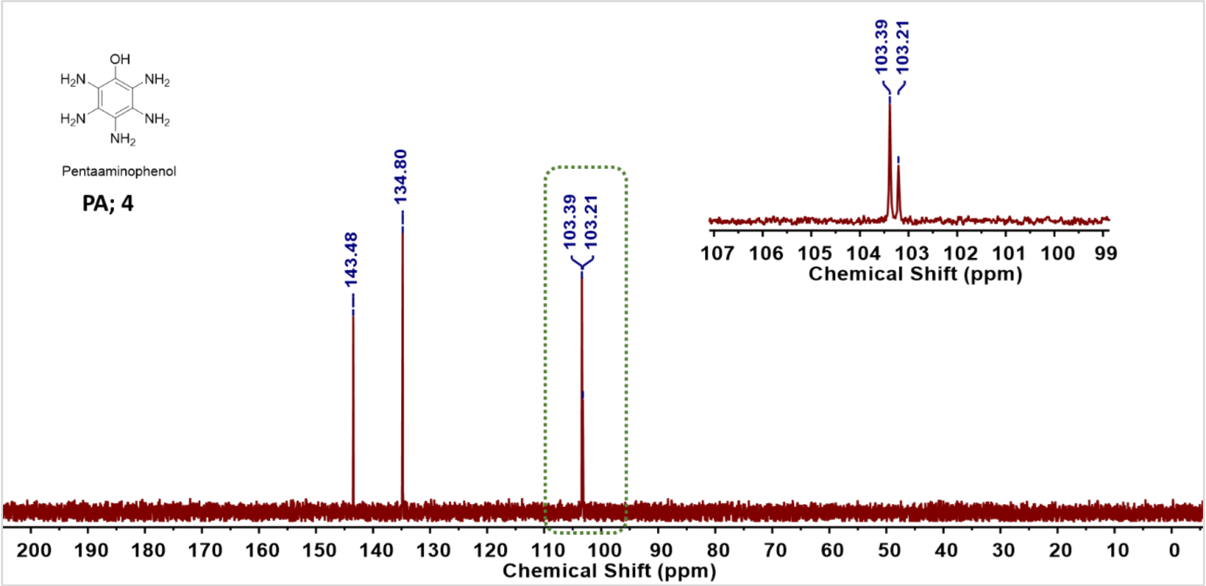

Supplementary Fig. 16. ^{13}C NMR spectrum of pentaaminophenol (PAP; **4**) (D_2O, 400 MHz).

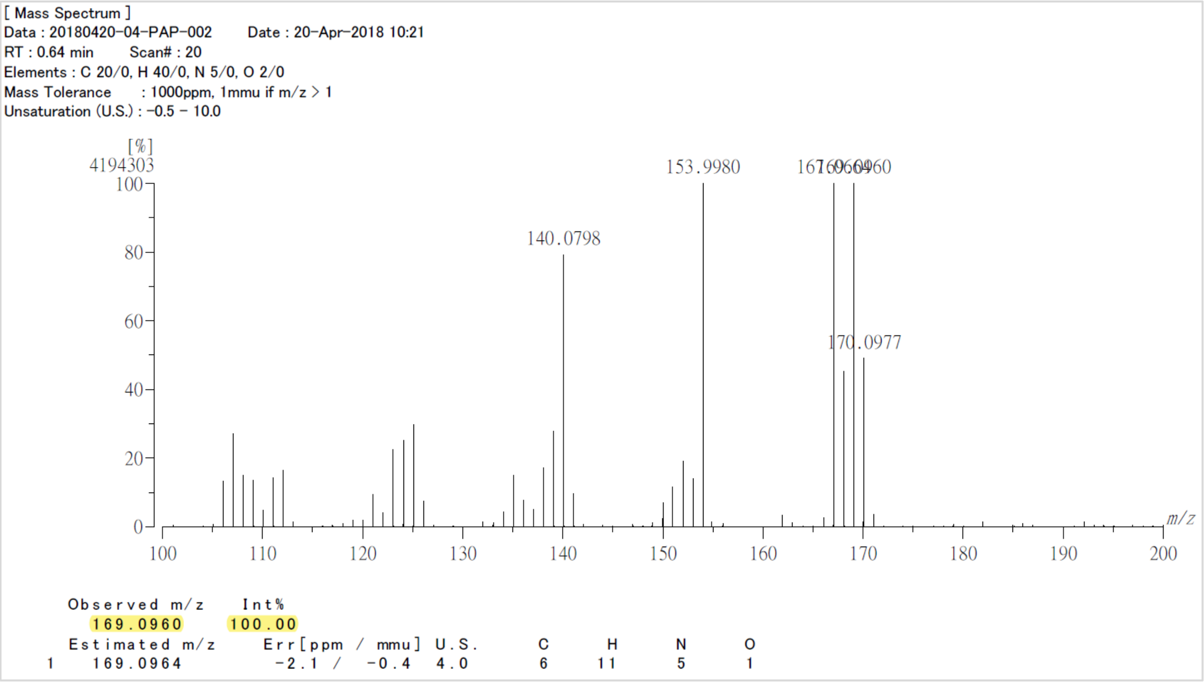

Supplementary Fig. 17. High-resolution mass spectrum (HR-MS) of pentaaminophenol (PAP; **4**).

Supplementary Table 1. Elemental analysis (EA*) from the F-COF

F-COF	Carbon	Hydrogen	Nitrogen	Oxygen
**Theoretical**	54.30	1.97	31.66	12.07
**Experimental**	55.14	2.14	28.61	13.85

*EA is most reliable technique for elemental counts for bulk sample.

Supplementary Note 1.

The broad XRD peaks are very common in the irreversible reaction driven organic framework synthesis. In solution with fast growing molecular weight because of large energy gain (aromatization), it is difficult to stack in a crystalline manner due to difficulty in the movement (kinetic) of the large flakes. The broad XRD peaks could also be related to poor crystallinity and small crystal size. Straight forward application of Scherrer equation, which is limited for nano-scale crystallites, for polymer structure is not very simple due to polycrystallinity. The shape of crystallites is usually irregular, most of the applications of the Scherrer analysis assume spherical crystallite shapes. The broadening can result from the non-uniform lattice distortions, dislocations, mixture of crystalline phases and grain boundaries. It is also suggested that the internal pressure exerted by the surface tension on the nanomaterial will create a stress field to trigger lattice strain, in case of small crystallite size.[1]

Supplementary Note 2.

Important note regarding STM: Thermal evaporation under UHV is possible in the STM chamber, but aftereffects are terrible. The flakes rigorously evaporate and sublimate in the chamber. To be frank, the sample preparation chamber remains contaminated with those molecules even after months. Baking procedure cannot completely remove the sublimized flakes attached to different parts of the chamber. Even after repeated baking of the system/chamber to remove the contaminants, the sample heating stage remains contaminated by these molecules, which evaporate from somewhere in the chamber during cleaning process. Thus, getting a clean surface even after repeated cycles of sputtering and annealing is difficult, because the contaminated sample stage becomes the source of those molecules during annealing. After cleaning the chamber many times (for a few weeks), sample stages become clean (but the molecules are reevaporated onto from somewhere inside the chamber again). As a result, getting a clean sample surface becomes very difficult. Hence, even if we bake the

chamber, sample stage becomes dirty again and again. We should spend 2~4 weeks to obtain a clean surface. It is really serious problem for STM study. Even replacing a chamber could not solve this problem, because not only the chamber but also every STM component is contaminated during the evaporation process.

Supplementary Methods

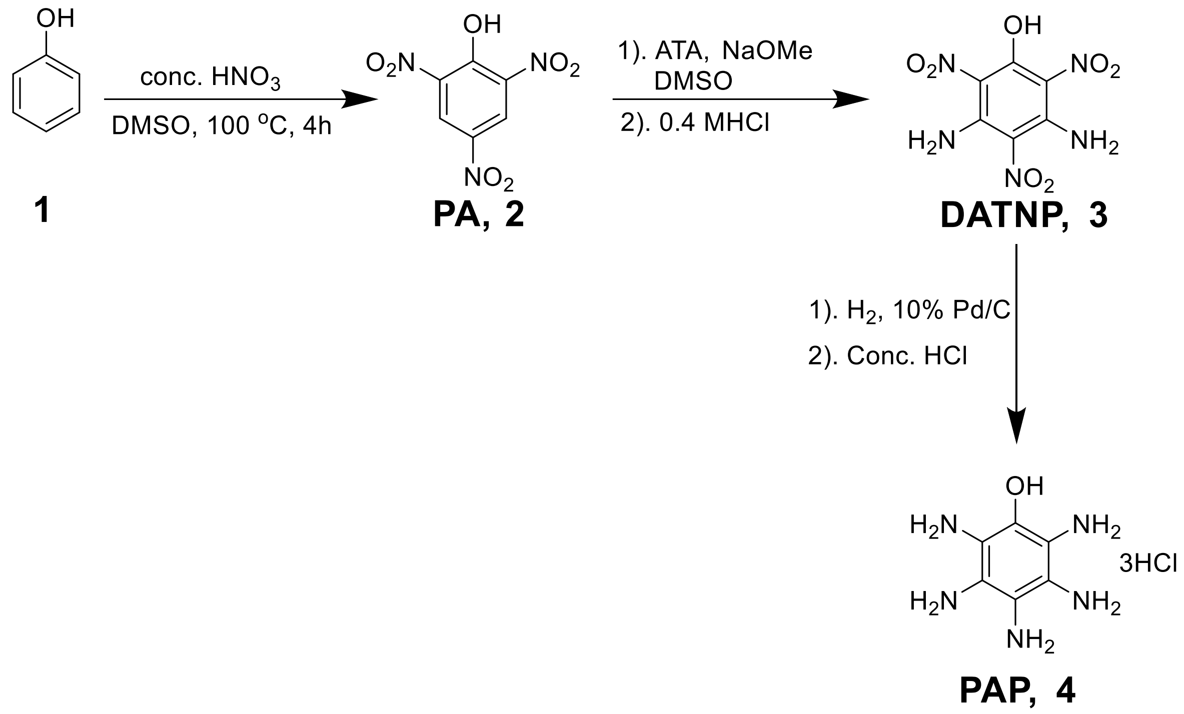

Synthesis of picric acid (PA, 2).[2] In a round-bottom flask, phenol (9 g, 95.4 mmol) was dissolved in dimethylsufoxide (DMSO, 18 mL) and then nitric acid (65%, 72 mL) was slowly added, while the reaction flask was cooling in an ice-water bath. After gentle stirring for 1 h, the light brown solution turned dark brown. It was further heated to 100 °C for 4 h. A deep brown gas evolved during the reaction, and then the color of the reaction mixture changed to yellow after further heating for 1.5–2 h. After completion of the reaction, the reaction mixture was cooled in an ice-water bath and poured into ice-cold water (400 mL). The precipitate was collected by suction filtration, washed with ice-cold water and air dried to produce yellow fine crystals (18 g, 82.19 % yield). Pure 2,4,6-trinitrophenol was obtained after recrystallization from benzene afforded as a yellow crystalline solid. mp. 121 °C (lit. mp. 122.5 °C); ^{1}H NMR: δ (400 MHz, MeOD) = 9.06 (s, 2H, CH aromatic), 4.99 (s, H, –OH) ppm (**Supplementary Fig. 11**). ^{13}C NMR δ (400 MHz, MeOD) = 153.08 (C-NH_2), 139.99 (C-NO_2), 138.95 (C-NO_2), 126.27 (C-H aromatic) ppm (**Supplementary Fig. 12**). Anal. Calcd. for $C_6H_3N_3O_7$: C, 31.46;

H, 1.32; N, 18.34; O, 48.88. Found: C, 31.33; H, 1.32; N, 18.22; O, 48.51%. HRMS calcd for $C_6H_3N_3O_7$ 228.9971; Found: 228.9962 (**Supplementary Fig. 13**).

Synthesis of 3,5-diamino-2,4,6-trinitrophenol (DATNP, 3).[3] In a round-bottom flask, sodium methoxide (MeONa, 23.8 g, 0.44 mol) was added to a solution containing PA (4.56 g, 0.02 mol) and 4-amino-1,2,4-triazole (ATA) (16.8 g, 0.2 mol) in DMSO (300 mL). The reddish-orange suspension was stirred at ambient temperature for 3 h. The reaction mixture was then poured into cold aq. HCl (0.4 M) solution. The resulting precipitates were collected by suction filtration, washed with distilled H_2O and dried. The solid was dissolved in DMSO in the presence of small NaOH and the solution was gently heated to 70 °C. When the product was completely dissolved in the DMSO solution, it was poured into ice-cold aq. HNO_3 (0.4 M) solution. The product precipitate was again collected by suction filtration and dried to give a bright yellow powder (4.62 g, 89% yield) of TATB. mp >300 °C (dec.) [lit.2 >300 °C (dec.)]. Due to poor solubility, acquiring NMR spectra was difficult. Anal. Calcd (%) for $C_6H_5N_5O_7$ (259.13): C, 27.81; H, 1.94; N, 27.03; O, 43.22. Found: C, 27.78; H, 1.99; N, 26.98; O, 43.77. HRMS calcd for $C_6H_5N_5O_7$ 259.0189; Found: 259.0179 (**Supplementary Fig. 14**).

Synthesis of 2,3,4,5,6-pentaaminophenol (PAP, 4). In a high-pressure hydrogenation vessel, 3,5-diamino-2,4,6-trinitrophenol (DATNP) (3.0 g, 12 mmol) and anhydrous ethyl acetate (150 mL) were placed. Palladium (10 %) on activated carbon (Pd/C, 0.5 g) was added. The reaction mixture was agitated under hydrogen (H_2) atmosphere (60 psi) until the yellowish colored DATNP suspension completely disappeared. Then, concentrated HCl (100 mL) was added into the reaction vessel and further agitated under H_2 (60 psi) for an additional 5 h.[3] The reaction mixture was then filtered under reduced pressure over Celite to remove the Pd/C catalyst. PAP trihydrochloride was nicely crystallized out. The crystals were further washed with 2M aq. hydrochloric acid (HCl) and dried to give quantitative yield.

To further purify and obtain high-quality single crystals, the white crystals (2 g) were dissolved again in deionized and degassed water (10 mL), filtered through a PTFE membrane (0.45 μm) to remove solid impurities, if any, and concentrated HCl (70 mL) was added. The flask was tightly sealed and placed in a freezer until very large needle-type crystals fell out. PAP trichloride exhibits no melting point but becomes dark and decomposes above 220 °C. ^{1}H NMR: δ (400 MHz, D_2O) = 4.70 (s, -NH_2, -OH) ppm (**Supplementary Fig. 15**). ^{13}C NMR δ (400 MHz, D_2O) = 143.48 (C-OH), 134.80 (*para*-C-NH_2), 103.39 (*ortho*-C-NH_2), 103.21 (*meta*-C-NH_2) ppm (**Supplementary Fig. 16**). Anal. Calcd (%) for $C_6H_{14}Cl_3N_5O$ (278.56): C, 25.87; Cl, 38.18; H, 5.07; N, 25.14; O, 5.74 Found: C, 25.75; H, 5.18; N, 25.03; O, 8.15; (Cl, 35.86). HRMS calcd for $C_6H_{11}N_5O$ 169.0964; Found: 169.0960 (**Supplementary Fig. 17**).

Supplementary References

1 Bindu, P. & Thomas, S. Estimation of lattice strain in ZnO nanoparticles: X-ray peak profile analysis. *J. Theor. Appl. Phys.* **8**, 123-134 (2014).

2 Khabarov, Y. G. *et al.* One-step synthesis of picric acid from phenol. *Org. Prep. Proced. Int.* **49**, 178-181 (2017).

3 Mahmood, J., Kim, D., Jeon, I.-Y., Lah, M. S. & Baek, J.-B. Scalable synthesis of pure and stable hexaaminobenzene trihydrochloride. *Synlett* **24**, 246-248 (2013).
